# Autonomous Parameter Adjustment for SSVEP-Based BCIs with a Novel BCI Wizard

**DOI:** 10.3389/fnins.2015.00474

**Published:** 2015-12-22

**Authors:** Felix Gembler, Piotr Stawicki, Ivan Volosyak

**Affiliations:** Faculty of Technology and Bionics, Rhine-Waal University of Applied SciencesKleve, Germany

**Keywords:** brain-computer interface, brain-machine interface, steady-state visual evoked potential, SSVEP, speller, BCI illiteracy, BCI deficiency, BCI inefficiency

## Abstract

Brain-Computer Interfaces (BCIs) transfer human brain activities into computer commands and enable a communication channel without requiring movement. Among other BCI approaches, steady-state visual evoked potential (SSVEP)-based BCIs have the potential to become accurate, assistive technologies for persons with severe disabilities. Those systems require customization of different kinds of parameters (e.g., stimulation frequencies). Calibration usually requires selecting predefined parameters by experienced/trained personnel, though in real-life scenarios an interface allowing people with no experience in programming to set up the BCI would be desirable. Another occurring problem regarding BCI performance is BCI illiteracy (also called BCI deficiency). Many articles reported that BCI control could not be achieved by a non-negligible number of users. In order to bypass those problems we developed a SSVEP-BCI wizard, a system that automatically determines user-dependent key-parameters to customize SSVEP-based BCI systems. This wizard was tested and evaluated with 61 healthy subjects. All subjects were asked to spell the phrase “RHINE WAAL UNIVERSITY” with a spelling application after key parameters were determined by the wizard. Results show that all subjects were able to control the spelling application. A mean (SD) accuracy of 97.14 (3.73)% was reached (all subjects reached an accuracy above 85% and 25 subjects even reached 100% accuracy).

## 1. Introduction

Brain-Computer Interfaces (BCIs) transfer electroencephalographic (EEG) brain signals collected by non-invasive electrodes and elicited by the user into computer commands, without using the brain's normal output pathways of peripheral nerves and muscles (Wolpaw et al., 2002). There are different control paradigms for such BCIs. Among the most common approaches are the steady-state visual evoked potential (SSVEP)-paradigm (Müller-Putz et al., 2005; Bin et al., 2009; Gao et al., 2014), the event-related desynchronization/synchronization (ERD/ERS)-paradigm (Blankertz et al., 2007), and the P300 event-related potential (ERP)-paradigm (Townsend et al., 2010). Usability challenges have impeded BCI usage in everyday scenarios for a long time. Recently this issue has been addressed by various research groups. Millán et al. (2010) predicted in their review that the time is ripe for developing practical BCI prototypes that will have a real impact in improving life quality of disabled people. Since then more effort has been made to conduct studies with the target population (Sellers et al., 2010; Holz et al., 2013; Kaufmann et al., 2013; Riccio et al., 2013; Daly et al., 2015; Kübler et al., 2015). For example, Sellers et al. (2010) tested a BCI used by a 51-year-old ALS-patient at his home. The system was used successfully for over 2.5 years and restored the user's independence in social interactions. Recalibration was performed remotely (via the internet). Holz et al. (2013) also installed a BCI controlled application at a locked-in ALS-patient's home. Their study demonstrated expert-independent home-use of BCI but it also reported varying performance and technical problems and stresses the importance of regular calibration.

BCIs are seeing considerable research interest, as there has been consistent growth in papers mentioning BCI from 2001 to 2012 (Thompson et al., 2014). PubMed search results with the search term “Brain-Computer Interface/Brain-Machine Interface” provide over 4200 results for articles in the last decade, and with the additional search term “SSVEP” there are still 185 results. The focus on this paper lies exclusively on SSVEP-based BCIs, which have become quite common in the meantime and represent a standard BCI paradigm. Visual stimuli (e.g., a set of boxes flickering at different constant frequencies) can be displayed simultaneously and independently on a computer monitor. By gazing at a particular box the user can select a desired command. Through, looking at a stimulus, brain signals are modulated with the corresponding frequency. Measured by an EEG the brain signals can then be classified in real time. Therefore, various applications like spelling interfaces (Volosyak et al., 2011a) and control applications for a prosthesis (Müller-Putz and Pfurtscheller, 2008) or for navigation (Martinez et al., 2007) can be implemented with the SSVEP approach. As SSVEP-based BCI depends on gaze shifting, these systems may not work for severely disabled people. However, SSVEP-based BCIs have also been tested with handicapped users (see Volosyak et al., 2009a; Müller et al., 2015). A SSVEP BCI system relies on a variety of different parameters which influence the BCI performance, such as stimulation frequencies and classification thresholds. For example, it has been observed that stimulation with lower stimulation frequencies, yields larger amplitudes (Gao et al., 2003; Zhu et al., 2010; Inkaew et al., 2015) and that the SSVEP response is maximum at 15 Hz (Pastor et al., 2003). Those need to be adjusted precisely. Therefore, automatized calibration methods are an essential step for BCIs to progress from laboratory demonstrators to real live applications, as the precise parameter set up cannot be expected from users or caretakers and caregivers.

Calibration methods have already become the standard for ERP-based BCIs. Typically, in a supervised classifier, data is collected during a calibration phase in which the user is asked to perform specific tasks. The collected data of brain signals is then analyzed and decoded to customize control parameters. Many research articles concentrate on shortening or omitting the calibration periods and on the development of so called Zero Training BCIs (Krauledat et al., 2008; Grizou et al., 2013; Kindermans and Schrauwen, 2013; Kindermans et al., 2014). Also Kaufmann et al. (2012) developed a user-centered ERP-BCI application that adjusts classifier weights and control parameters individually in the background and demonstrated feasibility of auto-calibrating ERP-BCI use. A wizard that handles important parameters especially for the SSVEP-paradigm was suggested by Volosyak et al. (2010b). Punsawad and Wongsawat (2012) proposed a SSVEP-based BCI system that requires less assistance from the caretaker, as it could be enabled or disabled by alpha band EEG, but to our best knowledge an automated calibration process for SSVEP-based BCIs has yet not been developed. The calibration software presented here was tested and evaluated with 61 subjects. We further explored BCI demographics based on the data of this large number of subjects. Previous BCI field-studies have been made with other non-invasive BCI approaches like the P300 paradigm (e.g., Guger et al., 2009) and the motor imagery paradigm (e.g., Guger et al., 2003). But except for Guger et al. (2012) the field-studies focusing on the SSVEP paradigm report subjects that were not able to gain satisfactory control over the BCI (e.g., Allison et al., 2010; Volosyak et al., 2011b).

Subjects are referred to as BCI-illiterates, it the BCI software cannot detect their intentions accurately, more precisely, if the classification accuracy cannot surpass a certain threshold (see e.g., Dornhege, 2007; Blankertz et al., 2010; Brunner et al., 2010; Fernandez-Vargas et al., 2013); e.g., the value of 70% is often used in the literature (Perelmouter and Birbaumer, 2000; Brunner et al., 2010). Therefore, we define the BCI literacy rate as the percentage of users who are able to achieve control over the BCI and the BCI illiteracy rate analogously. It should be noted that those terms (e.g., both BCI illiteracy and synonymously used BCI deficiency) have been criticized for being pejorative; those terms imply that it is the BCI end-user's “fault” that he or she cannot control the BCI. Yet, one could argue that it is not the fault of the user but of the BCI software that has not been able to perform proper classifications. Therefore, some authors prefer the use of the term “lack of BCI efficiency” instead (e.g., Vidaurre et al., 2011). However, the term BCI efficiency has already been defined in a different manner as the proportion of minimum number of compulsory commands to the total number of detected commands (Volosyak et al., 2009b). Therefore, we decided to use the regular historically established term “BCI illiteracy.” Though, some improvements in the BCI software algorithms are clearly visible over the years, the “BCI illiteracy” phenomenon remains a reoccurring problem in SSVEP-BCI field-studies. Allison et al. (2010) reported a BCI illiteracy rate of 24.52%, Volosyak et al. (2009b) mentioned 13.51% and Volosyak et al. (2011b) an illiteracy rate of 2.33%.

The overall aim of this research was

- to investigate optimal stimuli selection for SSVEP-based BCIs through analysis of the wizard outputs,
- to show that the vast majority, if not all BCI users are able to control a SSVEP-based BCI application, and
- to prove that generally higher classification accuracies can be achieved (through autonomous parameter adaption by the wizard and changes in the signal classification algorithms).

The used classification methods are based on the algorithms developed in our previous studies (e.g., Volosyak et al., 2009b, 2011b; Allison et al., 2010). In these studies the topic of inter-subject variability has been addressed as well. In order to compare the BCI performance, we further analyzed whether factors such as gender and sleep influence performance. For the analysis of such demographic factors all participants of the study went through the same questionnaires as in the mentioned publications.

## 2. Methods and materials

### 2.1. Hardware

The subjects were seated in front of a LCD screen (BenQ XL2420T, resolution: 1920 × 1080 pixels, vertical refresh rate: 120 Hz) at a distance of about 60 cm. The computer system used operated on Microsoft Windows 7 Enterprise and was based on an Intel processor (Intel Core i7, 3.40 GHz). Standard Ag/AgCl electrodes were used to acquire the signals from the surface of the scalp. The ground electrode was placed over *AF*_*Z*_, the reference electrode over *C*_*Z*_, and the eight signal electrodes were placed at predefined locations on the EEG-cap marked with *P*_*Z*_, *PO*_3_, *PO*_4_, *O*_1_, *O*_2_, *O*_*Z*_, *O*_9_, and *O*_10_ according to the international system of EEG electrode placement (Oostenveld and Praamstra, 2001). Standard abrasive electrolytic electrode gel was applied between the electrodes and the scalp to help bring impedances below 5 kΩ. An EEG amplifier g.USBamp (Guger Technologies, Graz, Austria) was used. The sampling frequency was set to 128 Hz. During the EEG signal acquisition, an analog band pass filter between 2 and 30 Hz and a notch filter around 50 Hz were applied directly in the amplifier.

### 2.2. Wizard

**Figure 1:**
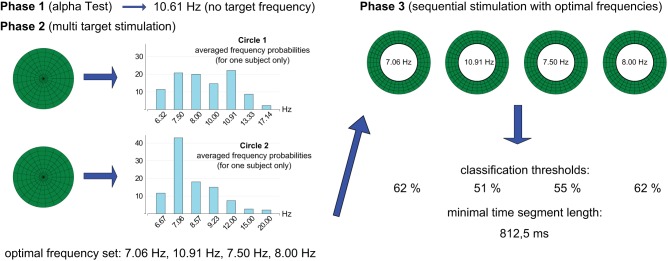
**Illustration of the calibration procedure for one subject**. After EEG-data were recorded (subject's eyes closed) 10.61 Hz had the strongest SSVEP response and was determined as alpha wave frequency. As this frequency did not belong to the set of possible target frequencies no frequency was filtered out. In phase 2 (multi-target stimulation) EEG-data were recorded while the subject faced two circles in sequence (each for 10 s). The first circle presented the target frequencies 6.32, 7.50, 8.00, 10.00, 10.91, 13.33, and 17.14 Hz. The second circle presented 6.67, 7.06, 8.57, 9.23, 12.00, 15.00, and 20.00 Hz. Results from the EEG recordings are displayed in the diagrams. In phase three, frequencies with the highest SSVEP-responses from both recordings were presented in series, and corresponding SSVEP key parameters were calculated.

The wizard ran the user through three phases in order to provide subject-specific stimulation frequencies (phase 1 and 2), classification thresholds, and time segment lengths (phase 3). The techniques used in each step were derived from several previous findings. The so called multi-target technique for the selection of individual subject-dependent stimulation frequencies, presented by Volosyak et al. (2010c), was based on the dual stimulation technique suggested by Mukesh et al. (2006) that used frequency combinations in order to increase the number of SSVEP targets. Figure 1 illustrates the entire calibration procedure for one subject.

The first task of the wizard was to select four optimal stimulus frequencies. One of the reasons for choosing a low-frequency band for SSVEP visual stimulation, were larger amplitudes (Gao et al., 2003; Zhu et al., 2010; Inkaew et al., 2015). Because of this, the wizard took only frequencies between 6.32 and 20 Hz into consideration. Furthermore, the number of suitable frequencies on the LCD monitor is limited due to the vertical refresh rate of 120 Hz. The stimulating frequencies have to be the sub-frequencies of the monitor refresh rate (Volosyak et al., 2009a; Chen et al., 2014; Nakanishi et al., 2014). Therefore, the optimal frequencies were drawn from the set of fourteen possible target frequencies 6.32, 6.67, 7.06, 7.50, 8.00, 8.57, 9.23, 10.00, 10.91, 12.00, 13.33, 15.00, 17.14, and 20.00 Hz (obtained with dividers between 6 and 24, see Table 1).

**Table 1:** **Overview of the potential target stimuli (dividers of the monitor refresh rate 120 Hz)**.

**Divider**	**19**	**18**	**17**	**16**	**15**	**14**	**13**	**12**	**11**	**10**	**9**	**8**	**7**	**6**
Frequencies	6.32	6.67	7.06	7.50	8.00	8.57	9.23	10.00	10.91	12.00	13.33	15.00	17.14	20.00

#### 2.2.1. Classification method

For SSVEP signal classification we used a minimum energy combination method (MEC) introduced by Friman et al. (2007), as modified by Volosyak (2011). To detect a frequency in the spatially filtered signals the SSVEP power estimations for all *N*_*f*_ frequencies were normalized into probabilities

(1) $$\begin{array}{r} p_{i}\;=\;\frac{\hat{P_{i}}}{\sum_{j=1}^{N_{f}}{\hat{P}}_{j}}\;\;\text{with}\;\;\overset{N_{f}}{\underset{i=1}{\sum }}p_{i}=1, \end{array}$$

where $\hat{P_{i}}$ is the *i*th power estimation, 1 ≤ *i* ≤ *N*_*f*_.

To increase robustness in the four target spelling application we considered three additional frequencies, selected as means between two target frequencies (see also Volosyak et al., 2010a).

The classifier output *O* was the index of the *i*th frequency if the following conditions held: (1) the *i*th frequency had the highest probability $p_{i}^{\prime }$, (2) $p_{i}^{\prime }$ exceeded certain predefined thresholds β_*i*_, and (3) the detected frequency belonged to one of the stimulating frequencies. So for a BCI system with four targets the output was defined as

(2) $$\begin{array}{l} O=\left\{ \begin{array}{c} {\text{argmax}}_{i}\left(p_{i}^{\prime }\right)\;\\ p_{i}^{\prime }\geq \beta_{i}\;\\ i\leq 4,\; \end{array}\right. \end{array}$$

where 1 ≤ *i* ≤ *N*_*f*_. If no frequency probability exceeded the corresponding threshold β_*i*_ or if one of the additional frequencies had highest probability the output *O* was set to zero. The choice of the β_*i*_ depended on the corresponding stimulation frequency (generally lower stimulation frequencies produce higher SSVEP-response, therefore thresholds can be higher) but also on user factors (the quality of the SSVEP-signal differs between subjects). The values for the β_*i*_ were determined in a calibration session with the here presented wizard. If *O* was classified as an additional frequency (*i* > 4), then the classification would be rejected as the detected frequency did not belong to the set of stimulation frequencies.

EEG-data were processed by the computer in blocks of 13 samples (101.5625 ms with the used sampling rate of 128 Hz). Therefore, the time segment lengths displayed in Table 2 were used. The classification was performed with the sliding window of *T*_*s*_ after receiving the new EEG data block.

**Table 2:** **Overview of the used time segment lengths**.

**Segment-length**	**Time (ms)**	**Blocks of EEG data (one block = 13 samples)**
*T*_1_	812.5	8 Blocks
*T*_2_	1015.625	10 Blocks
*T*_3_	1523.4375	15 Blocks
*T*_4_	2031.25	20 Blocks
*T*_5_	3046.875	30 Blocks
*T*_6_	4062.50	40 Blocks
*T*_7_	5078.125	50 Blocks
*T*_8_	6093.75	60 Blocks
*T*_9_	7109.375	70 Blocks
*T*_10_	8125	80 Blocks

*Ten segment lengths T_s_, between 812.5 and 8125 ms were used*.

#### 2.2.2. Phase 1 (alpha-test)

Whether high alpha wave activity occurred, was tested in the first phase of the wizard, as the low frequency band overlaps with the alpha band (8–13 Hz Zhu et al., 2010), which can cause false classifications (Zhu et al., 2010; Cao et al., 2014). Therefore, the frequencies from the set of possible target frequencies (see Table 1), which belonged to the alpha band, were checked for interference with each subject's alpha wave. Therefore, in the first phase of the wizard it was tested whether high alpha wave activity occurred.

When the wizard program was started, the user was instructed by an audio instruction and a text message displayed on the screen to close his or her eyes. After 10 s a second audio command told the user to open his or her eyes again. During the closed eye period EEG data were recorded. The five stimulation frequencies *f*_*st*_ = 8.57, 9.23, 10.00, 10.91, 12.00 Hz (possible target frequencies on the LCD monitor belonging to the alpha band) and ten neighboring frequencies *f*_*st*_±0.3 Hz were tested for SSVEP response. If after 10 s one of the possible target frequencies had the highest averaged probability and surpassed a certain threshold, it would be neglected further on. After this procedure, all of the remaining frequencies differed from the alpha wave by 0.15 Hz or more.

In all phases, signal-to-noise ratio (SNR) distributions for 10 different time segment lengths were calculated online. The criterion for frequency selection in phase 1 and phase 2 was based on the calculation of the integral value of SNR distribution of the different segment lengths over time.

Values for $p_{i}^{\prime }$ were calculated for all used time segment lengths and for all frequencies, so after 812.5 ms (eight blocks), a value for $p_{i,812.5ms}^{\prime }$ was calculated. As we recorded 100 blocks of EEG-data, 93 values (100−8+1 blocks) were calculated for $p_{i,T_{1}}^{\prime }$. After 10 s, those 93 values were summed and an average value ${\hat{p}}_{i,T_{1}}^{\prime }$ was calculated. This was done for all time segment lengths, so after 10 s we had averaged probabilities ${\hat{p}}_{i,T_{s}}^{\prime }$ for all ten used time segment lengths. Then the average over all ${\hat{p}}_{i,T_{s}}^{\prime }$ was calculated

(3) $$\begin{array}{l} {\hat{p}}_{i}^{\prime }=\frac{{\hat{p}}_{i,T_{1}}^{\prime }+{\hat{p}}_{i,T_{2}}^{\prime }+\ldots +{\hat{p}}_{i,T_{10}}^{\prime }}{10}. \end{array}$$

In phase 1 we considered 15 possible alpha frequencies (*N*_*f*_ = 15). The criteria for classifying the *i*th frequency as alpha frequency were (1) the *i*th frequency had the highest averaged probability ${\hat{p_{i}}}^{\prime }$ and (2) ${\hat{p_{i}}}^{\prime }>0.1$.

#### 2.2.3. Phase 2 (multi-target stimulation)

In phase 2, multi-target stimulation was used to find optimal stimulation frequencies (frequencies with the strongest SSVEP response). The user faced a circle (radius 245 pixels) divided into 147 segments (seven rings, each containing 21 segments) representing seven stimulating frequencies at once. Each of the seven stimulating frequencies was presented by 21 segments which were scattered randomly around the circle.

When subjects were instructed (by an audio message) to focus their gaze on the circle, the flickering started and EEG data were collected. After 10 s (100 blocks of EEG-data), the flickering paused for 2 s. Thereafter, the user faced a second circle, identical to the first one, but now it flickered with seven different frequencies. EEG data were recorded for another 10 s. Each circle contained higher and lower frequencies and, in order to avoid mutual influences between stimulating frequencies, each group of seven simultaneously flickering segments followed the additional restrictions rules (as e.g., in Volosyak et al., 2010c):

(4) $$\begin{array}{l} f_{i}\neq \left[f_{j}+f_{k}\right]∕2,f_{i}\neq 2f_{j}-f_{k},f_{i}\neq 2f_{k}-f_{j}. \end{array}$$

The stimulation frequencies for the first circle were 6.32, 7.50, 8.00, 10.00, 10.91, 13.33, and 17.14 Hz. For the second, they were 6.67, 7.06, 8.57, 9.23, 12.00, 15.00, and 12.00 Hz. If one of the 14 possible target frequencies interfered with the users alpha wave, this frequency would be neglected and one of the circles would contain only six frequencies. The probabilities Equation (3) of seven stimulation frequencies (*N*_*f*_ = 7) for each circle were calculated after data had been recorded. After this, the 14 possible target frequencies were sorted from highest averaged probability to lowest and the top four frequencies were selected as optimal target frequencies. However, the restriction rules Equation (4) were checked for each frequency from the set of those four selected frequencies in descending order and if they were violated, the corresponding frequency would be replaced with the frequency which had the highest averaged probability of the remaining frequencies. For example, if 6 Hz had the highest and 12 Hz the second highest averaged probability, the latter was replaced, because otherwise the restriction rules would be violated, as 12 Hz is a harmonic of 6 Hz.

#### 2.2.4. Phase 3 (sequential stimulation)

In order to find optimal thresholds for the SSVEP-classification a white circle (radius 150 pixels), flickering at the frequency which had the highest SSVEP response in phase 2, was displayed. It was necessary to simulate noise caused by peripheral vision when concentrating on the target object. Therefore, the white circle (the target object) was surrounded by a green ring (outer diameter 500 pixels, inner diameter 300 pixels), containing 144 segments. Each of the remaining three frequencies from the optimal frequency set was presented by 48 flickering segments which were scattered randomly around this ring. The user was instructed by an audio command to gaze at the white circle.

The circle and the ring flickered for 10 s while EEG data were recorded. The flickering then paused for 2 s so that further recordings would not be influenced by the SSVEP-responses that occurred during the first recording. Thereafter, the white circle flickered with the second highest frequency from phase two, while the ring flickered with the remaining three frequencies. This procedure was repeated until data for all four optimal frequencies were collected, so the total recording time for phase three was 40 s. Table 3 shows the blinking sequence of the four optimal frequencies during phase 3.

**Table 3:** **Blinking sequence during phase 3 (assuming that ***f***_**1**_, ***f***_**2**_, ***f***_**3**_, ***f***_**4**_ are the four optimal frequencies)**.

**Target frequency (white circle)**	**Frequencies contained in the “noise ring”**	**Flickering duration (s)**
*f*_1_	*f*_2_, *f*_3_, *f*_4_	10
*f*_2_	*f*_1_, *f*_3_, *f*_4_	10
*f*_3_	*f*_1_, *f*_2_, *f*_4_	10
*f*_4_	*f*_1_, *f*_2_, *f*_3_	10

After this, the classifier outputs *O* (see Equation 2) were analyzed. Let β = [β_1_, β_2_, β_3_, β_4_] be the vector of all four classification thresholds. Classification thresholds were chosen equal for all four frequencies (β_*i*_ = β_*j*_ for *i, j* = 1, 2, 3, 4). Classifier outputs *O*_*i*,_*T*__*s*_, β_ were determined for all $p_{i,T_{s}}^{\prime }$ and for all thresholds β, with β_*j*_ = 0.15, 0.16, …, 0.99 for *j* = 1, …, 4. The output was then categorized into three classes:

- If the output *O*_*i*,_*T*__*s*_, β_ was equal to the index of the stimulation frequency, the output was classified as “correct classification.”
- If *O*_*i*,_*T*__*s*_, β_ was equal to the index of one of the remaining three stimulation frequencies, the output was classified as “wrong classification.”
- If *O*_*i*,_*T*__*s*_, β_ was equal to zero (no frequency probability exceeded the thresholds β_*i*_ or an additional frequency had highest probability) the output was categorized as “zero classification.”

Note that there were only four stimulation frequencies in each circle but three additional frequencies were considered (*N*_*f*_ = 7). Then the distributions of correct classifications, *p*_*correct*_(*i*, β, *T*_*s*_), false classifications, *p*_*false*_(*i*, β, *T*_*s*_) and zero classifications, *p*_*zero*_(*i*, β, *T*_*s*_) for each of the four stimulation frequencies (*i* = 1, 2, 3, 4) was calculated.

The thresholds and minimal time segment lengths were selected in an iterative process: First all *O*_*i*,_*T*__*s*_, β_ for the *i*−*th* frequency were analyzed for the smallest time segment length *T*_*s*_ = 812.15 ms. The largest value β_*j*_ = 0.30, 0.31, …, 0.70, β = [β_*j*_, β_*j*_, β_*j*_, β_*j*_] which satisfied the conditions

(5) $$\begin{array}{l} p_{correct}\left(i,\beta ,T_{s}\right)\geq 40\text{ and }p_{false}\left(i,\beta ,T_{s}\right)=0 \end{array}$$

was determined and, if such value existed, the threshold β_*i*_ corresponding to the *i*th frequency was set to this value. This was carried out for each frequency individually. If β_*j*_ satisfying Equation (5) were found for all four stimulation frequencies, the minimal segment lengths *T*_0_ was set to *T*_*s*_ and the thresholds β_*i*_ were chosen as classification thresholds. If such β_*j*_ did not exist for at least one of the four stimulation frequencies, *T*_*s*_ was set to the next higher segment length and the conditions Equation (5) were checked again. This procedure was repeated until such β_*j*_ satisfying Equation (5) were found. Table 4 shows an example of the threshold determination process.

**Table 4:** **Distributions of classifier outputs ***p***_***correct***_, ***p***_***zero***_, and ***p***_***false***_ for a fixed frequency ***i*** and a fixed time segment length ***T***_***s***_**.

**β_*j*_ [%]**	***p*_*correct*_ [%]**	***p*_*zero*_ [%]**	***p*_*false*_ [%]**
30	97,53	0	2,47
31	96,30	0	3,70
⋮	⋮	⋮	⋮
50	44,44	0	55,56
**51**	**43,21**	**0**	**56,79**
52	39,51	0	60,49
⋮	⋮	⋮	⋮
69	23,46	0	76,54
70	20,99	0	79,01

*In this example the threshold for the corresponding frequency was set to 51 (bold), as it was the largest threshold β_j_ that satisfied equation (5). For all β_j_ > 51 (light gray) Equation (5) was not satisfied*.

### 2.3. Three-step spelling application

**Figure 2:**
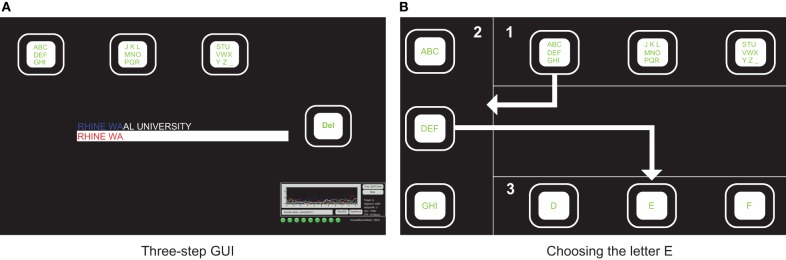
**GUI of the ***Three-step spelling application*****. Initial screen containing the alphabet in three flickering boxes **(A)**. An overview of the three steps necessary to choose a single letter **(B)**.

The *Three-step spelling application* (Gembler et al., 2014) resembles an earlier developed graphical user interface (GUI) layout (Volosyak et al., 2011a; Kick and Volosyak, 2014). The initial screen is displayed in Figure 2A. Four commands were represented on the computer screen by flickering boxes of default sizes (125 × 125 pixels). The size of the boxes varied during the experiment as described by Volosyak (2011). After selecting a desired box, the position of the three boxes containing the alphabet changed from upper horizontal to left-hand vertical according to the first selection made (see Gembler et al., 2014). After the second selection the positions changed once more from left vertical to horizontal bottom position. Also, each box now contained a single letter. In the second and the third step, the far right box (“Del” in the first step) would contain the command “back,” giving the user the option to switch to the previous view. An overview of the three steps necessary to choose a single letter is shown in Figure 2B.

Every command classification was followed by an audio feedback in order to reduce the information load on the visual channel.

**Figure 3:**
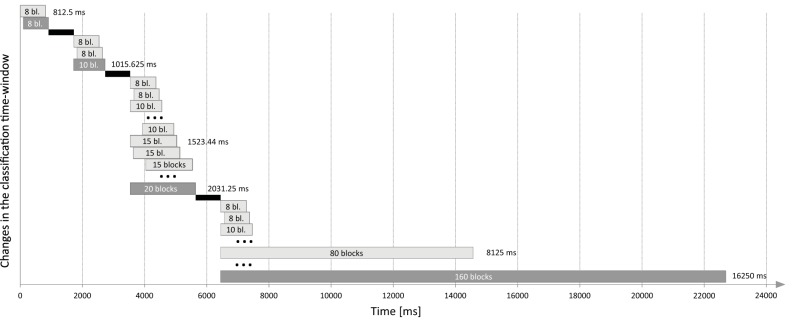
**Changes in the time segment length in case no distinct classification can be made at the moment and the actual time ***t*** allows the extension of the classification time-window to the next pre-defined value (e.g., 1015.625 ms after 812.5 ms)**. After each classification (gray), additional time for gaze shifting was included (black) and the classifier output was rejected for nine blocks.

The SSVEP classification was performed on the basis of the adaptive time segment length of the acquired EEG data (Volosyak, 2011). If no classification could be made and the actual time *t* allowed the extension of *T*_*s*_ to the next predefined value, this new value was used instead (see Figure 3). The fixed starting segment length was determined by the wizard software. We further included a segment length of 160 blocks (16 s, see also Gembler et al., 2015).

### 2.4. Subjects

All subjects (healthy adult volunteers) gave written informed consent in accordance with the Declaration of Helsinki. This research was carried out in accordance to best practice guidelines; ethical principles were taken into consideration during conducting of all BCI experiments. Information needed for the analysis of the experiments was stored anonymously during the experiment; results cannot be traced back to the participant. Sixty-one subjects participated, with a mean (SD) age of 22.8 (3.89) years (range 17–49); 17 of the subjects were female. All subjects were students or employees of the Rhine-Waal University of Applied Sciences. The EEG recording took place in a standard laboratory room with low background noise and luminance. None of the subjects had neurological or visual disorders. Spectacles were worn when appropriate. Subjects did not receive any financial reward for participating in this study.

### 2.5. Procedure

After completing the consent form, each subject completed a brief pre-questionnaire, where questions regarding gender, the need for vision correction, tiredness, and BCI experience were answered. Thereafter, subjects were prepared for the EEG recording. At first subjects went through the steps of the Wizard software and key parameters for BCI performance were determined. Once started by the experimenters, subjects were guided by audio and text feedback through the phases and used the wizard independently. The key parameters found by the wizard were transferred automatically to the *Three-step spelling application* which was then started by the experimenters. Subjects participated in a familiarization run spelling the word “BRAIN” and a word of choice (e.g., the own first name). Next, each subject used the GUI to spell the phrase “RHINE WAAL UNIVERSITY.” The spelling phase ended automatically when the phrase was spelled correctly. The experiment would have been stopped manually in case a subject had not been able to execute a desired classification within a certain time frame, had wished to end the experiment, or if unintentional repeated misclassifications had occurred. However, every subject was able to complete the spelling task. Spelling errors were corrected via the implemented delete button. Information needed for the analysis of the test was stored anonymously during the experiment. After the spelling phase the subjects completed a post-questionnaire, answering questions regarding tiredness and their subjective opinion about the BCI system.

### 2.6. ITR calculation

The information transfer rate (ITR) represented the amount of information communicated per unit time and was calculated based on the following formula (Wolpaw et al., 2002):

(6) $$\begin{array}{l} B=\log_{2}N+P\log_{2}P+\left(1-P\right)\log_{2}\left[\frac{1-P}{N-1}\right]. \end{array}$$

In the formula above, *B* represents the number of bits per trial. The Accuracy *P* was calculated as the ratio between the number of correct selections and the total number of classified commands. The number of possible choices was the number of flickering boxes available (*N* = 4). To obtain ITR in bits per minute, *B* is multiplied by the number of command classifications per minute. ITR and accuracy were calculated on-line and displayed at the lower right corner of the GUI of the *Three-step spelling application* during the spelling task (see Figure 2A).

## 3. Results

### 3.1. Spelling performance

All 61 subjects were able to complete the spelling task; no subject reported any pain or discomfort during the experiment. Table 5 shows the overall spelling performance for the spelling task from 61 subjects.

**Table 5:** **Results of spelling the phrase “RHINE WAAL UNIVERSITY”**.

**Subject**	**Time (*s*)**	**Acc (%)**	**ITR (bpm)**	**Subject**	**Time (*s*)**	**Acc (%)**	**ITR (bpm)**
1	262.95	98.51	28.48	34	253.81	98.46	28.81
2	351.71	97.01	20.07	35	481.71	88.00	11.89
3	537.86	95.65	12.85	36	831.39	89.39	9.66
4	505.07	98.51	14.83	37	359.02	87.95	17.64
5	633.34	97.01	11.15	38	256.55	100.00	29.47
6	422.50	91.01	17.90	39	311.39	98.46	23.28
7	559.31	92.00	11.79	40	321.95	95.89	22.91
8	307.94	98.51	24.32	41	242.73	100.00	31.15
9	264.47	100.00	28.59	42	249.44	100.00	30.30
10	916.40	100.00	8.25	43	558.59	95.65	12.37
11	346.73	100.00	21.80	44	595.97	95.71	11.79
12	204.14	100.00	37.03	45	260.51	100.00	29.02
13	277.98	100.00	27.20	46	222.22	100.00	34.02
14	425.55	97.01	16.59	47	484.35	94.67	14.97
15	395.18	100.00	19.13	48	261.63	97.01	26.98
16	857.70	94.67	8.45	49	294.73	100.00	25.65
17	364.51	98.46	19.89	50	312.71	100.00	24.18
18	230.75	100.00	32.76	51	299.41	100.00	25.25
19	246.70	100.00	30.65	52	301.03	100.00	25.11
20	427.38	100.00	17.69	53	517.87	85.32	14.65
21	486.08	97.01	14.52	54	221.31	100.00	34.16
22	670.21	98.46	10.82	55	273.00	92.59	26.66
23	297.17	100.00	25.44	56	226.48	100.00	33.38
24	433.77	89.89	16.76	57	240.70	97.01	29.33
25	369.99	97.01	19.08	58	456,83	98,46	15,87
26	202.41	100.00	37.35	59	338.51	97.01	20.85
27	411.94	95.89	17.91	60	409.91	90.91	15.90
28	403.51	98.51	18.56	61	517.56	94.67	14.01
29	299.91	100.00	15.21
30	361.56	92.96	17.86	Min	202.41	85.32	8.25
31	324.09	100.00	23.33	Max	916.40	100.00	37.35
32	403.51	91.36	17.26	Mean	383.65	97.02	21.58
33	424.23	97.10	17.19	SD	155.40	3.71	7.52

*All subjects were able to complete the task. Mean values are given at the bottom of the table*.

The analysis of the spelling performance reveals an overall mean (SD) ITR of 21.92 (7.63) bpm and a mean (SD) accuracy of 97.14 (3.73)%. All Subjects reached 85–100% accuracy; 24 of the 61 subjects even completed the spelling task without errors, achieving an accuracy of 100%.

### 3.2. Wizard

**Figure 4:**
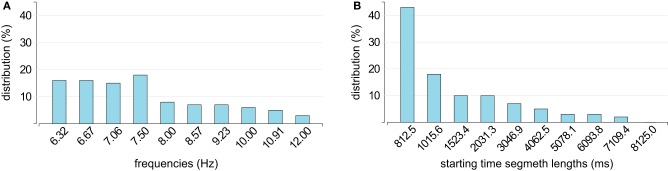
**Distribution of (A) stimulation frequencies and (B) lengths of the starting time window over all subjects determined by the wizard**.

The SNR distributions for the stimulation in two groups of seven frequencies (multi-target stimulation) were analyzed with the Minimum Energy Combination algorithm (Section 2.2.1) to find the best four stimulation frequencies. Figure 4A shows for how many subjects each frequency was selected (wizard phase 2). The data were analyzed with the Minimum Energy Combination algorithm to find the best four stimulation frequencies.

The optimal time segment for each user was selected in a similar way. In phase two, selected stimuli were presented in sequence and again the SNR distributions were analyzed with the Minimum Energy Combination algorithm and were used to calculate the accuracy for each segment length and classification border simultaneously. Based on these values, a minimal time segment length was chosen. Figure 4B shows how often each time segment length was chosen.

The length of the time-window used for Minimum Energy Combination method was 10 s for each calibration step of the wizard. Thus, the wizard returns adequate outcomes from data sets recorded during 70 s (10 s for phase one, 20 s for phase two, and 40 s for phase three).

### 3.3. Questionnaire results

In the pre-questionnaire subjects answered questions regarding gender, the need for vision correction, tiredness, and BCI experience, as displayed in Table 6. Seventeen subjects (27.9%) were female [22.71 (4.29) years] and 72% were male subjects [22.83 (5.22) years]. Female subjects performed with an information transfer rate of 25.35 (6.54) bpm while males performed with a rate of 20.12 (7.34) bpm. A *t*-test revealed a significant difference between the mean ITR of female and male subjects, *t*_(32)_ = 2.64, *p* < 0.05. The results of the post-questionnaire are given in Table 7.

**Table 6:** **Results from the pre-questionnaires**.

**Age**	**Gender**		**Vision correction**		**Level of tiredness**					**Length of sleep last night**	**Experience with BCIs**	
**Years**	**m**	**f**	**Yes**	**No**	**(1)**	**(2)**	**(3)**	**(4)**	**(5)**	**h**	**Yes**	**No**
22.8 (5.02), 17–49	44	17	22	39	19	25	15	3	0	6.76 (1.19), 4–9	6	55

*The numbers are represented as number of respondents or in form: mean value (SD), range. The level of tiredness was rated on a scale from 1 to 5: (1), not tired; (2), little tired; (3), moderately tired; (4), tired; and (5), very tired*.

**Table 7:** **Results from the post-questionnaires as number of respondents**.

**Level of tiredness**					**Flickering annoying**		**Recommend BCI to others**	
**(1)**	**(2)**	**(3)**	**(4)**	**(5)**	**Yes**	**No**	**Yes**	**No**
17	20	20	1	3	20	41	58	3

*The level of tiredness was rated on a scale from 1 to 5: (1), not tired; (2), little tired; (3), moderately tired; (4), tired; and (5), very tired*.

## 4. Discussion

The presented wizard for SSVEP-based BCI sets up key parameters in adequate time and allows inexperienced personnel to set up the BCI, as only one click is necessary for calibration. Overall, including pauses between steps, the calibration process took <2 min. However, a calibration that runs in the background, invisible to the user as already developed for the P300-paradigm (see e.g., Kaufmann et al., 2012) is desirable for the SSVEP-paradigm as well. Regarding user friendliness, almost all subjects stated that they would recommend the system, though nearly a third of the subjects stated to be annoyed by the flickering. This is in line with observations from other studies (see e.g., Müller et al., 2015). In their review Zhu et al. (2010) also summed up the disadvantages of using lower frequencies. Subjects tend to be more annoyed by lower frequencies, visual fatigue occurs more easily, stimuli can provoke epileptic seizures, and the low frequency band covers the alpha band.

In the presented study, 14 subjects reported slightly increased tiredness (see Tables 6, 7). However, five subjects reported a decrease in tiredness. Thus, for the majority of subjects the calibration and spelling performance with the SSVEP-based BCI did not produce significant fatigue. As also reported by Volosyak et al. (2011b), neither the hours of sleep nor the subjective level of tiredness influenced BCI performance. However, in the presented study the effect of gender was significant. A trend that female subjects might perform better with lower frequencies was also observed in previous works (Allison et al., 2010; Volosyak et al., 2011b). This observation needs further investigation.

As has been discussed intensively throughout the BCI literature, a major challenge in SSVEP-based BCIs lies in finding a compromise between accuracy and speed. The choice of the time-window dedicated to the SSVEP response detection during EEG analysis has high impact on the accuracy. While a short time-window results in classification errors, a longer time-window slows performance down (Volosyak et al., 2010a; da Cruz et al., 2015). As the commands corresponding to the stimulating frequencies are produced only if their probabilities are higher than certain predefined thresholds, classification thresholds are another important factor in finding balance between speed and accuracy. The wizard determined these variables specifically for each user. Figure 4B shows a wide variety of ideal minimum time segment lengths among the 61 subjects. The highest minimal segment length determined was 7109.4 ms but in most cases the shortest possible minimal time segment length of 812.5 ms was selected. Though, a longer minimal time segment length results in lower ITR, it yields higher accuracies, and for some users a long time-window at the beginning was necessary to guarantee control over the BCI system. The system might have been unable to interpret intentions for those users if fixed segment lengths were used. For the majority of subjects, lower frequencies were selected (see Figure 4A). For nine subjects the lowest possible frequency set 6.32, 6.67, 7.06, and 7.50 Hz was determined, and although the frequencies are separated by < 0.5 Hz, this set worked well. This observation is in harmony with the observation by Gao et al. (2003), that two flickering targets with a frequency difference as low as 0.2 Hz can be successfully distinguished in the SSVEP response. This value has recently been updated to 0.1 Hz (Hwang et al., 2012). Lower stimulation frequencies can even be distinguished with a difference of 0.05 Hz (Stawicki et al., 2015). The most frequently selected frequency was 7.5 Hz, which might be explained by the fact that that its second harmonic is 15 Hz, which is the stimulation frequency at which the SSVEP response is maximum according to Pastor et al. (2003). As lower frequencies overlapping with the alpha band (8–13 Hz), might cause false classifications (Zhu et al., 2010; Cao et al., 2014). Otherwise, simple closing of the eyes might lead to false classifications. Therefore, the frequencies were checked for interference with each subject's alpha wave in the first phase of the wizard software.

In our previous SSVEP-based field-studies the BCI literacy rate could steadily be improved and now, after more than 5 years of research, 100% has been achieved. Allison et al. (2010) reported 80 out of 106 and Volosyak et al. (2009b) 32 out of 37 subjects that were able to perform a spelling task. Due to further modifications BCI illiteracy rate could be reduced to 2.33% (Volosyak et al., 2011b). Only Guger et al. (2012) showed that their SSVEP-BCI could provide effective communication for all 53 subjects. One simple cause for a high literacy rate in SSVEP-based BCIs is a low number of stimulation targets. Guger et al. (2012) as well as Volosyak et al. (2011b) also used four simultaneously displayed stimulation frequencies (see Table 8). It should be noted that BCI literacy among all participants was achieved in studies using other BCI approaches as well. Kaufmann et al. (2012) reported that all 19 subjects were able to complete a spelling task with a P300 speller with an average accuracy of 91.2% and an ITR of 15.1 bpm and in a study with 99 subjects Guger et al. (2003) reported a BCI literacy rate of 100% as well. Guger et al. (2009) also achieved full BCI literacy with 81 subjects using the motor imagery paradigm.

**Table 8:** **Comparison of BCI performance (mean accuracies) of different SSVEP-BCI field-studies**.

	**Volosyak et al., 2009b**	**Volosyak et al., 2011a**	**Guger et al., 2012**	**Presented study**
Number of subjects	37	86	57	61
Mean accuracy (%)	92.9	92.3	95.5	97.1
Literacy rate (%)	86.5	97.7	100	100
Number of classes	5	4	4	4
Classification time-window (s)	2	2	3	0.8-8

*In the first two studies (2009 and 2011) all BCI illiterate subjects were excluded from further calculation of mean values*.

Further we would like to point out, that generally higher ITRs than in the presented study can be achieved with BCIs. For example, Spüler et al. (2012) reported an average ITR of 144 bpm and an accuracy of 96% with a BCI that used code-modulated, visual evoked, potentials and the detection of error-related potentials. Although, the ITR of 21.92 (7.63) bpm in the here presented study is considerably low compared to these values, the results are promising because of the very high mean (SD) accuracy of 97.14 (3.73)% which slightly surpasses the values from previous field-studies using four stimuli classes. In comparison Volosyak et al. (2011b) reported that subjects reached an accuracy of 92.26 (7.82)%, but two subjects were unable to achieve control over the system. LEDs were used as stimulation source and subject navigated a miniature robot through a labyrinth. As in the presented study four distinct commands were used. In a previous smaller sized study, the same graphical user interface as in the presented study was tested with six healthy subjects and a mean accuracy of 87.41 (6.74)% was reached (Gembler et al., 2014). Frequencies and time segment lengths were not selected user dependently. In comparison the mean (SD) accuracy of 97.14 (3.73)% achieved in the presented study is significantly larger [*t*_(5)_ = 3.44, *p* < 0.05], which supports our hypothesis, that the amount of misclassifications can be reduced through automated user-specific parameter selection and larger classification time-windows. Guger et al. (2012) also used a relatively large classification time-window and achieved a BCI literacy rate of 100% (see Table 8).

According to Perelmouter and Birbaumer (2000) a typical patient's estimation “almost absolutely reliable” is equivalent to a classification accuracy value between 90 and 95%. Almost all subjects (93.44%) in this study surpassed 90% classification accuracy and 77.05% of the subjects achieved accuracies above 95%.

### Limitations

On rare occasions (four subjects) the determined classification threshold for one of the frequencies was too low and a test subject performed poorly during the familiarization run. This could be explained by diminishing concentration of the subject during the calibration process, or by the fact that the wizard did not take the spatial arrangement of the boxes into account, since it always concerned the corresponding box “del,” which had rather prominent position. In these cases, the calibration process was repeated. Therefore, further software improvements are necessary.

It should also be mentioned that for long term use recalibration might be inevitable and that the here presented SSVEP-based BCI depends on the user's vision and control over the eye movements. The wizard usually selected lower frequency sets as the determination is based on the SSVEP-response. However, visual stimulation with low frequencies is known to cause fatigue.

Furthermore, subjects in this study may not be reflective of the general population; they tended to be young healthy men, therefore additional tests with older and physically impaired people are needed.

## 5. Conclusions

A wizard for a SSVEP-based BCI that automatically determines individual BCI parameters for each user and can be handled by inexperienced personnel has been tested with 61 healthy subjects. The presented study confirms that through careful user-specific choice of SSVEP BCI constants such as stimulation frequencies, classification thresholds, and segment lengths, high accuracies can be achieved by a broad population.

All subjects achieved reliable control over the BCI system, reaching accuracies above 85%. A comparison with previous field-studies proves, that the introduced modifications are an essential step to broaden the literacy rates of BCI systems to all potential users. The main causes for the achieved literacy rate and high accuracies are:

- the number of simultaneously displayed targets is limited to four,
- subject specific frequency and threshold selection through the presented wizard, and
- extended classification time-windows (>8 s) for poor performers.

Further research might also consider other BCI paradigms. Volosyak et al. (2010b) introduced the BCI wizard as a system that automatically identifies key parameters to customize the best BCI paradigm for each user. Tailoring a BCI including the input signal has also been suggested by Guger et al. (2012); Kübler et al. (2014). Furthermore, the construction of the wizard software and its GUI allow an extension, so that key parameters could also be determined for BCIs with a higher number of stimuli. Because of the mentioned disadvantages of lower stimulation frequencies, the wizard could be modified, so that higher frequencies are also considered. The methods of the Wizard might be integrated directly in applications, such as the speller presented, so that spatial proximity of targets is also taken into account when selecting key parameters.

### Conflict of interest statement

The authors declare that the research was conducted in the absence of any commercial or financial relationships that could be construed as a potential conflict of interest.
